# Microglia: The breakthrough to treat neovascularization and repair blood-retinal barrier in retinopathy

**DOI:** 10.3389/fnmol.2023.1100254

**Published:** 2023-01-23

**Authors:** Xuefei Fu, Shuyu Feng, Huan Qin, Lin Yan, Caiyan Zheng, Kai Yao

**Affiliations:** ^1^Institute of Visual Neuroscience and Stem Cell Engineering, Wuhan University of Science and Technology, Wuhan, China; ^2^College of Life Sciences and Health, Wuhan University of Science and Technology, Wuhan, China; ^3^Hubei Province Key Laboratory of Occupational Hazard Identification and Control, Wuhan University of Science and Technology, Wuhan, China

**Keywords:** microglia, retinopathy, retinal neovascularization, blood-retinal barrier, retinal inflammation

## Abstract

Microglia are the primary resident retinal macrophages that monitor neuronal activity in real-time and facilitate angiogenesis during retinal development. In certain retinal diseases, the activated microglia promote retinal angiogenesis in hypoxia stress through neurovascular coupling and guide neovascularization to avascular areas (e.g., the outer nuclear layer and macula lutea). Furthermore, continuously activated microglia secrete inflammatory factors and expedite the loss of the blood-retinal barrier which causes irreversible damage to the secondary death of neurons. In this review, we support microglia can be a potential cellular therapeutic target in retinopathy. We briefly describe the relevance of microglia to the retinal vasculature and blood-retinal barrier. Then we discuss the signaling pathway related to how microglia move to their destinations and regulate vascular regeneration. We summarize the properties of microglia in different retinal disease models and propose that reducing the number of pro-inflammatory microglial death and conversing microglial phenotypes from pro-inflammatory to anti-inflammatory are feasible for treating retinal neovascularization and the damaged blood-retinal barrier (BRB). Finally, we suppose that the unique properties of microglia may aid in the vascularization of retinal organoids.

## 1. Introduction

**Figure 1:**
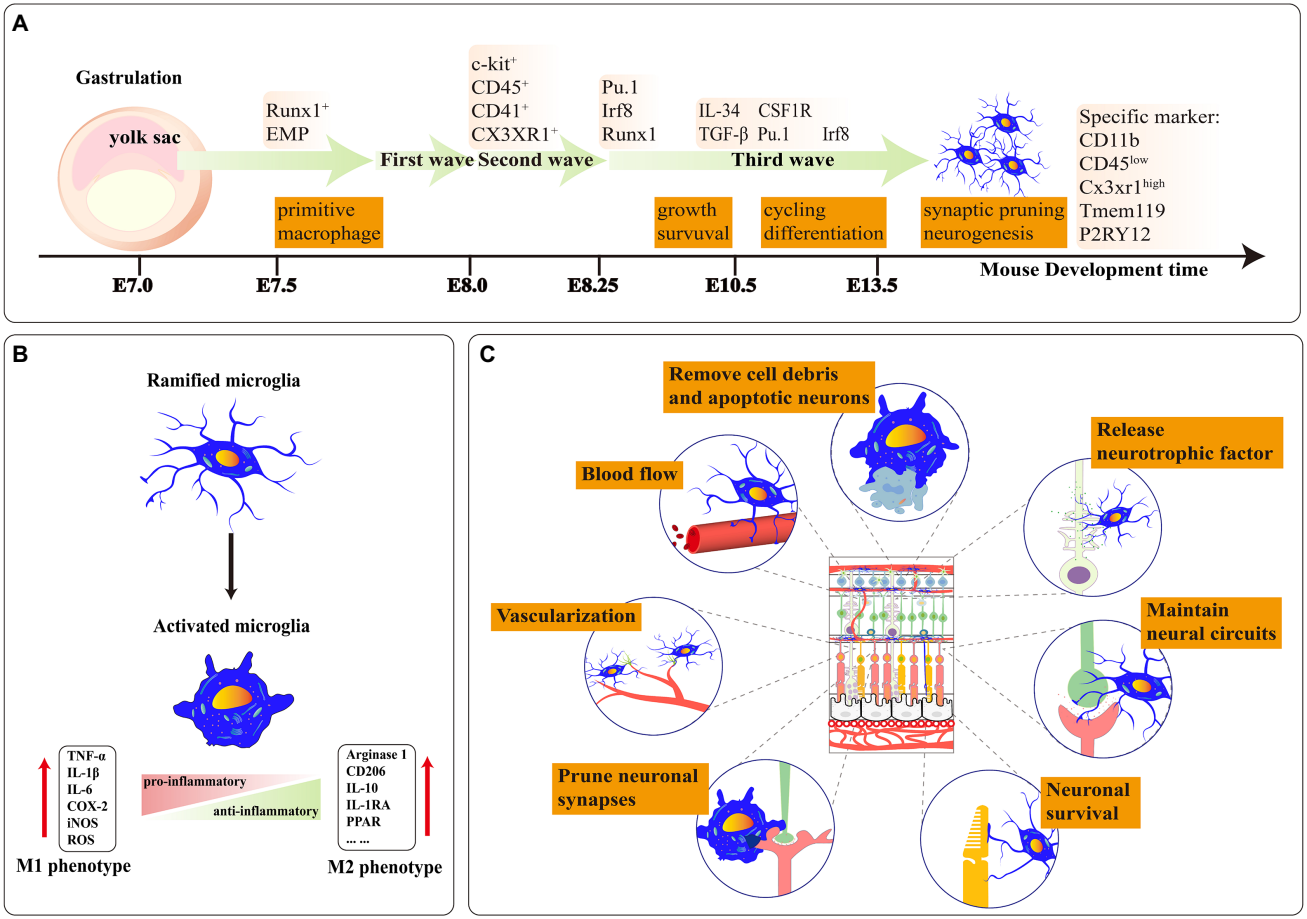
**(A)** A stepwise microglia developmental program in the retina. Mature microglia maintain a stable population derived from self-renewal. E-embryo. **(B)** Ramified microglia can be activated to two polarized phenotypes: M1 and M2. The polarization of M1 microglia increased the expression level of proinflammatory factors. Moreover, the polarization of M2 microglia expressed a high level of anti-inflammatory factors. **(C)** The functions of microglia in healthy retina tissue: Phagocytosis of apoptotic cells and debris; releasing neurotrophic factors to neurons; maintaining neural circuits; ensuring the survival of neurons; pruning neuronal synapses; promoting angiogenesis in retinal development; controlling the retinal vascular blood flow rate.

Microglia, which are derived from the yolk sac, are the primary resident mononuclear macrophages in the retina and are regarded as the first line of active immune defenders. They express many pattern recognition receptors and immune receptors, such as *AT1R*, *AT2R*, and *TβRII*, empowering their function of guiding blood vessel formation, guaranteeing the survival of neurons, monitoring neuronal activity, pruning synapses of neurons, sustaining density of dendritic spines and clearing apoptotic cells or protein aggregates (Takahashi et al., 2005; Parkhurst et al., 2013; Cramer et al., 2022; He et al., 2022; Figure 1). Retinal microglia mainly present high branch structures to guarantee retinal homeostasis. When the balance of the retinal microenvironment is broken, microglia change their phenotypes, such as amoeboid mode, and stay in an activated state. Activated microglia are one of the essential neurotoxic mediators of neuroinflammation (Deng et al., 2009; Zhao et al., 2015). They rapidly move to damage sites and secrete pro-inflammatory factors (e.g., *IL-1β*, *IL-6*, *TNF-α*), chemokines (e.g., *CCL2*, *CCL4*, *CXCL10*) resulting in secondary damage to neuronal death, thereby accelerating the disease progression of patients (Wu et al., 2021; Puthenparampil et al., 2022).

The retina, a highly active tissue member of the central nervous system, requires high oxygen consumption and metabolic demands. The retinal vascular systems transport nutrients to the retina and break down metabolites. In retinal diseases, such as proliferative diabetic retinopathy and retinopathy of prematurity, neovascularization exacerbate the loss of sight. Microglia participate in neovascularization and present co-localization with the neovascular plexus. According to recently published papers, researchers classified microglia into pro-inflammatory and anti-inflammatory based on the classification method of macrophages (Cano-Cano et al., 2021). The conversation from the pro-inflammatory microglia to the anti-inflammatory-microglia effectively reduces the formation of neovascularization (Zhou et al., 2021). In addition, endothelial cells cooperate with other types of cells and form the blood-retinal barrier (BRB) which strictly limits the exchange of various molecules and segregates the plasma and the tissue. Activated microglia promote the dysfunction of BRB which further aggravates plasma leakage and the death of cells. And it is possible that interventions targeting microglia could alleviate neovascularization and the damage to the blood-retinal barrier, thereby helping patients preserve their sight.

This review narrates the correlation of microglia with retinal neovascularization and the blood-retinal barrier. Targeting microglial activation may represent an attractive therapeutic method in retinopathy. Microglia involve in retinal vascularization during normal retinal development. Under some retinopathy, microglia participate in pathological angiogenesis, particularly in hypoxia conditions. The large number of inflammatory factors released by continuously activated microglia can accelerate the loss of vision and lead to the death of retinal endothelial cells, pericytes, retinal pigment epithelium (RPE), and neurons. Pro-inflammatory microglial death is fatal to the retinal disease process. Hence, we recapitulate three microglial signaling pathways we are more interested in. They may monitor retinal vascular function and decipher how microglia migrate near blood vessels through intercellular signaling crosstalk. This evidence demonstrates the vital role of microglia in the retinal vasculature and BRB. Finally, we summarize the research progress of microglia induced by iPSC (iMG) which is a reliable method to predict the molecular mechanisms of retinal microglia *in vitro*. And the unique properties of microglia may contribute to vascularizing human retinal organoids.

## 2. Retina microglia, retina vessel, and blood-retinal barrier

### 2.1. Microglia in retina

#### 2.1.1. Derivation and localization of retinal microglia

Yolk sac progenitor cells in gastrula ultimately evolve into retinal microglia through three developmental waves from differentiation to primitive bone marrow cells. At mouse E7.0–7.5, the yolk sac progenitor cells in the blood land specifically express the RUNX family transcription factor 1 (*Runx1*) and differentiate to C-kit^+^ early bone marrow erythroid progenitor cells. At mouse E8.0, bone marrow erythroid progenitor cells enter the first wave of growth and precisely express *CD41* and *CD45*. At E8.25, *CD41^+^* and *CD45^+^* cells access the second wave of development and further distinguish to the precursor microglia under the regulation of transcription factors such as SPi-1 proto-oncogene (*PU.1*), interferon regulatory factor (*Irf8*) and *Runx1*. At mouse E9.5, the precursor microglia enter the central nervous system (CNS) supported by Sodium/Calcium exchanger protein (*NCX-1*) and then colonize in the retina (Ginhoux et al., 2010; Prinz et al., 2021). At mouse E10.5, these cells eventually differentiate into the naive microglia under the stimulus factor of transforming growth factor beta (*Tgfβ*), colony stimulating factor 1 receptor (*CSF1R*), and interleukin 34 (*IL34*). EMR1/ADGRE1 (*F4/80^+^*) microglial population can be identified in the retina at E11.5 (Ajami et al., 2007; Tay et al., 2017). At mouse E12.5, the number of *F4/80^+^* cell populations steadily increases and then proliferates and migrates from the central area to the retinal periphery (Santos et al., 2008). At mouse E14.5, microglia possess the function of synaptic pruning and shaping neural circuits (Figure 1A).

#### 2.1.2. Microglial colonization and recolonization after ablation

Microglia progressively migrate to the whole retina during development and are finally located in ganglion cell layer (GCL), inner plexiform layer (IPL), outer plexiform layer (OPL), and nerve fiber layer (NFL) layers (Lee et al., 2008). Microglial *CSF1R* is one of the basic receptors to colonize the retina (Ginhoux et al., 2010). *CSF1R*, as a tyrosine kinase transmembrane receptor, has two activating ligands (Wei et al., 2010); (1) *CSF-1*, known as macrophage colony stimulating factor (M-*CSF*), plays an essential role in regulating the proliferation, differentiation, and survival of macrophages (Sherr et al., 1985); (2) *IL34*, mainly produced by glial cells and neurons, considers a substitute ligand for *CSF-1* (Wei et al., 2010). When *CSF-1* and *IL34* bind to *CSF1R*, they encourage non-covalent dimerization of the receptor chains and transphosphorylation of tyrosine residues (Yu et al., 2008). And *CSF-1* and *IL34* have different affinities for D2 and D3 protein domains from *CSF1R* (Stanley and Chitu, 2014). Furthermore, unlikely microglia in OPL, microglia depend on *IL-34* secreted by other neurons to localize in IPL and have the feature of assisting cone and rod in transferring chemical signals (Greter et al., 2012).

The neuronal regeneration capacity is limited in the damaged adult mammalian retina (Jorstad et al., 2017). Nevertheless, microglia repopulate the retina *via* individual mitosis or migration of the microglia/macrophage outside the retina to guarantee population quantity (Zhang et al., 2018). Huang et al. used PLX5622 to eliminate nearly all endogenous microglia in the retina of *Cx3cr1^+/GFP^* transgenic mice and discovered two microglial refilling ways: The epibiotic microglia of the optic nerve enter the retinal optic disc and then refill from the retinal center to retinal periphery; another microglial refill way derived from corpus ciliare/iris is in the opposite direction with above (Huang et al., 2018a). In addition, the newly recolonized microglia compulsively own similar phenotypes to the endogenous microglia in function, morphology and proliferation under the influence of the retina-tissue environment (Jin et al., 2017; McPherson et al., 2019).

#### 2.1.3. The classification of retinal microglia

Retinal neuronal activity can affect microglial phenotypes (Anderson et al., 2022). The polarization phenotypes of retinal microglia present two extreme states: pro-inflammatory (M1) and anti-inflammatory (M2; Figure 1B). M1-microglia stay in “Classical activation” which secretes high levels of *TNF-α*, *IL-1β*, *ROS*, etc. Retinal *IL-1β* is primarily expressed by microglia and adding small doses of exogenous *IL-1β* increases neuronal survival ability in the excitotoxic condition (Todd et al., 2019). Appropriate activation of M1 microglia also facilitates axonal regeneration in trauma sites (Kigerl et al., 2009). However, continued activation of M1 microglia leads to irreversible neuronal damage (Tang and Le, 2016). In RD10, activated infiltrating microglia continuously secreted *IL-1β* which further accelerated the degeneration of the rod (Zhao et al., 2015).

The microglial phenotypes are in dynamic alternation during the disease process. M2 microglia can be subdivided into “alternative activation” and “acquired deactivation,” hinging on the activation environment and stimulation factors (Walker and Lue, 2015; Siddiqui et al., 2016). Alternative activated M2 microglia are heavily linked with functions such as anti-inflammatory repair and extracellular matrix reconstitution. In contrast, acquired deactivated M2 microglia convert their phenotype in response to anti-inflammatory factors (e.g., *TGFβ1*, *IL-4*) from the environment (Caruso et al., 2020; Chen et al., 2022). M2 microglia increase phagocytosis of erythrocytes and tissue debris, which facilitates hematoma regression (Lan et al., 2017). M1 transformation to M2 microglia alleviates the degeneration of photoreceptors in the mouse model RD1 (Zhou et al., 2018; Figure 1B). The conversion of the M1/ M2 phenotype in the appropriate period of acute or chronic retinopathy may provide better therapeutic benefits.

However, with the discovery of the biomarker, retinal microglia likewise generated different subsets of species, and their classification should not be limited to M1 or M2 in the fully polarized state (Wieghofer et al., 2021). In the meanwhile, Liu et al., had pointed out that a proliferative retinopathy-associated subset of microglia presented to perivascular newborn tufts in the oxygen-induced model (Liu Z. et al., 2022). Therefore, a rational classification of these microglia subsets and naming them with specialized nomenclature is necessary.

### 2.2. Microglia correlation with retinal vasculature and BRB in retina

Microglial branches contact retinal blood vessels, secrete nutritional factors and angiogenic factors, control the apoptosis of pericytes and endothelial cells, and timely eliminate redundancy vessel debris which plat an outstanding significance to maintaining retinal function (Ritter et al., 2006; Jolivel et al., 2015; Harsing et al., 2021). They regulate vessel diameter and blood flow velocity through neurovascular coupling (Császár et al., 2021; Mills et al., 2021). Astrocyte and Müller cells are also members of neurovascular coupling. Astrocytes mainly contact the superficial vascular plexus and the regulation ability of Müller cells is principally limited in the intermediate vascular plexus in stable conditions (Biesecker et al., 2016).

IPL and OPL are the main oxygen-consuming layers (Yu and Cringle, 2001). In these layers, microglial branches co-locate with blood vessels which is sufficient to justify the importance of microglia to the retinal blood vessel and the BRB. Therefore, in this section, we briefly introduce the structure of retina and then summarize the relevance of microglia to the retinal neovascularization and BRB.

#### 2.2.1. Structure of retina

**Figure 2:**
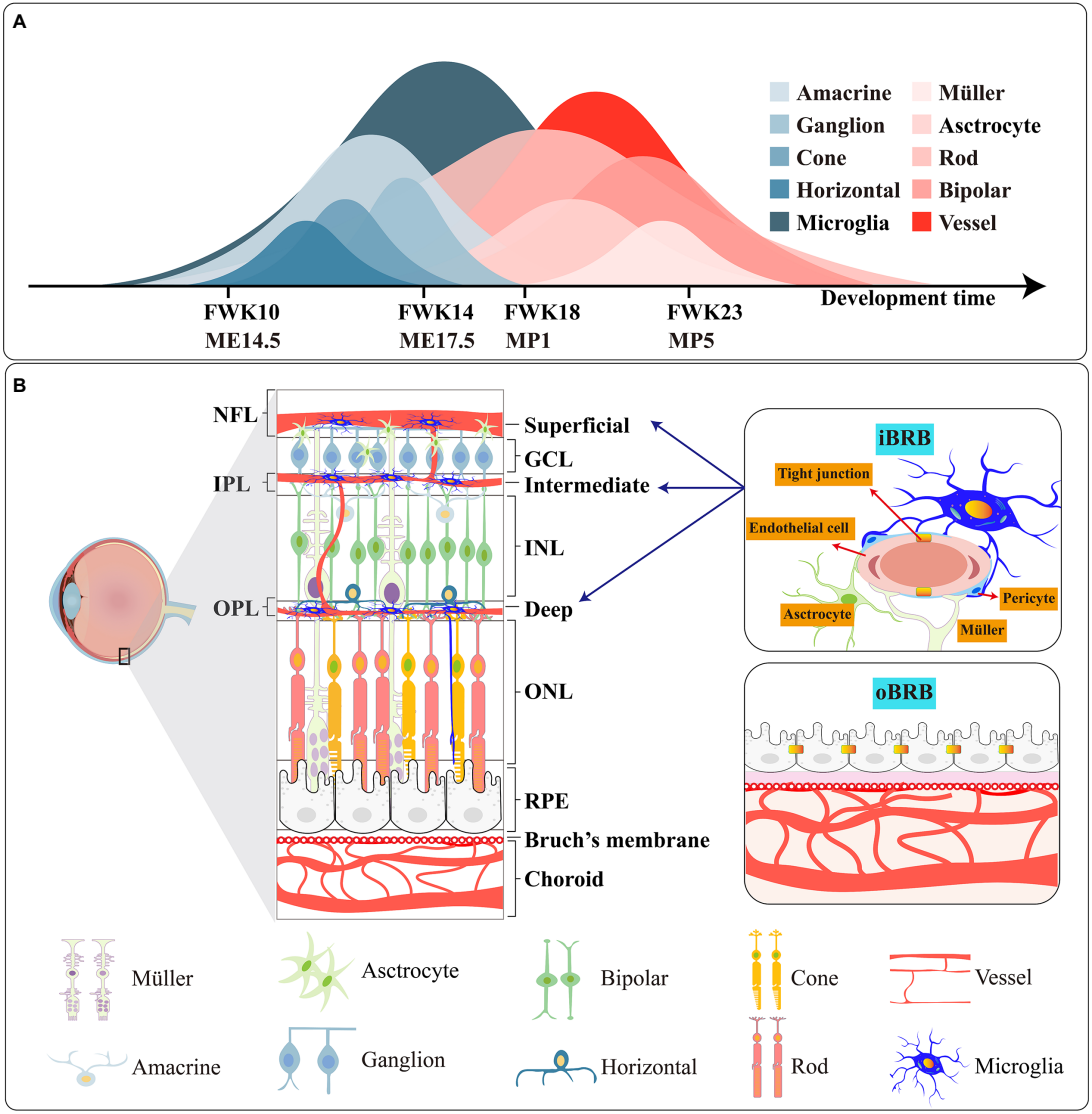
**(A)** Genesis of retinal cells during the development of the human and mouse. FWK-fetal week; ME-mouse embryo; MP-mouse postnatally. **(B)** Schematic picture of the retina, the iBRB and oBRB. The main types of tight junction protiens are different in iBRB and oBRB.

Retina, as a functional unit of the CNS, is mainly composed of six types of neurons (rod, cone, amacrine, bipolar, horizontal and ganglion) which convert light from the external environment into neural chemical signal and then transfer it to the brain through the optic nerve. And there are three species of glia cells; thereinto, astrocyte and Müller provide retina nutrition and supporting function. Microglia, as the third type of glia in the retina, supervises the homeostasis of the retinal environment in real time and resists foreign microorganism invasion. In structure, the retina is divided into: (1) ONL: cytons of cone and rod; (2) OPL: synapses of cones, rods and horizontal; (3) INL: cytons of bipolar, horizontal and amacrine and Müller; (4) IPL: synapses of bipolar, amacrine cells, retinal ganglion cells and Müller; (5) GCL: cytons of retinal ganglion cells (Figure 2).

#### 2.2.2. Retinal vasculature

The retina of humans, mice and rats have formed three kinds of vasculature systems in the long course of evolution. And the sequence of their development is choroidal vasculature - hyaloid vasculature-retinal vasculature (Lutty and McLeod, 2018). Retina, as a high oxygen consumption and metabolic demand tissue in the CNS (Kooragayala et al., 2015), has a dual blood supply vascular system: (1) Retinal vasculature: Central artery and vein radially extend along retinal exterior and then form arterioles and venules. Arterioles expand to the IPL and OPL layers and then continue to nurture capillaries. Blood backflow to venules along the capillaries eventually returns to the central vein and leaves the visual system through the optic nerve, and completes the blood cycle of vision. At 14 weeks of gestation in humans, the retinal vasculature starts to develop and enters the epilogue at 23 weeks (Hasegawa et al., 2008). Unlike humans, the retinal vasculature of the mouse comes to maturity about 1–2 weeks after postnatal development, and the time of maturation varies between strains (Stahl et al., 2010). After the development of the retinal vascular system, three layers of vascular plexus are differentiated in the GCL, IPL, and OPL, respectively called superficial vascular plexus, intermediate vascular plexus, and deep vascular plexus. And the growth rate of the deep is preferred to the middle (Fruttiger, 2007). (2) Choroid vasculature also consists of three blood plexuses. The outermost plexus is called Haller’s layer, the middle one is the Sattler layer, and the innermost anterior is the capillaries (Lutty and McLeod, 2018). The anterior capillaries allow plasma to congregate the surface of Bruch’s membrane, and vesicles in RPE deliver nutrients and oxygen from plasma to the rod and cone.

Pathological angiogenesis is the leading irreversible cause of blindness among potentially blinding eye diseases. Angiogenesis is a process defined as forming new blood vessels on basis of existing capillaries under the combined action of angiogenic factors and endothelial cells. Endothelial cells consolidate their proliferation and transferability under the action of vascular endothelial growth factor (*VEGF*), insulin like growth factor 1 (*IGF1*) and *Notch* family receptors and their ligands; some endothelial cells grow the filopodia and become endothelial tip cells (Gerhardt et al., 2003; Cao et al., 2017). Mature neovascularization connects with the initial vessels to participate in blood circulation and become a section of the vascular system (Zoya Tahergorabil, 2012). In multiple retinal diseases, such as RAP, the neovascularization extending to the avascular area aggravated vision loss in patients (Spaide, 2013). And the activated microglia are involved in angiogenesis during disease onset.

#### 2.2.3. BRB

BRB is vital to retinal function. Sharing resemblances with the blood–brain barrier in the brain, BRB protects the retina and isolates pathogens and microorganisms. The BRB consists of the inner blood-retinal barrier (iBRB) and the outer blood-retinal barrier (oBRB). Endothelial cells in the iBRB safeguard the integrity of the iBRB under the action of the neurovascular unit formed by microglia, Müller cells, astrocytes and pericytes (O'Leary and Campbell, 2021). And the oBRB is composed of choroid, Bruch’s membrane and RPE (Figure 2B).

In angiodynamic researches, fluorescence angiography (VFA) is commonly utilized to examine the half-rise, half-fall and offset time of blood flow filling in retinal arteries, veins and capillaries after intravenous injection of drugs to check the damage of the blood-retinal barrier, vascular leakage and angiogenesis (Hui et al., 2014).

#### 2.2.4. Microglial relationship with retinal vasculature

**Figure 3:**
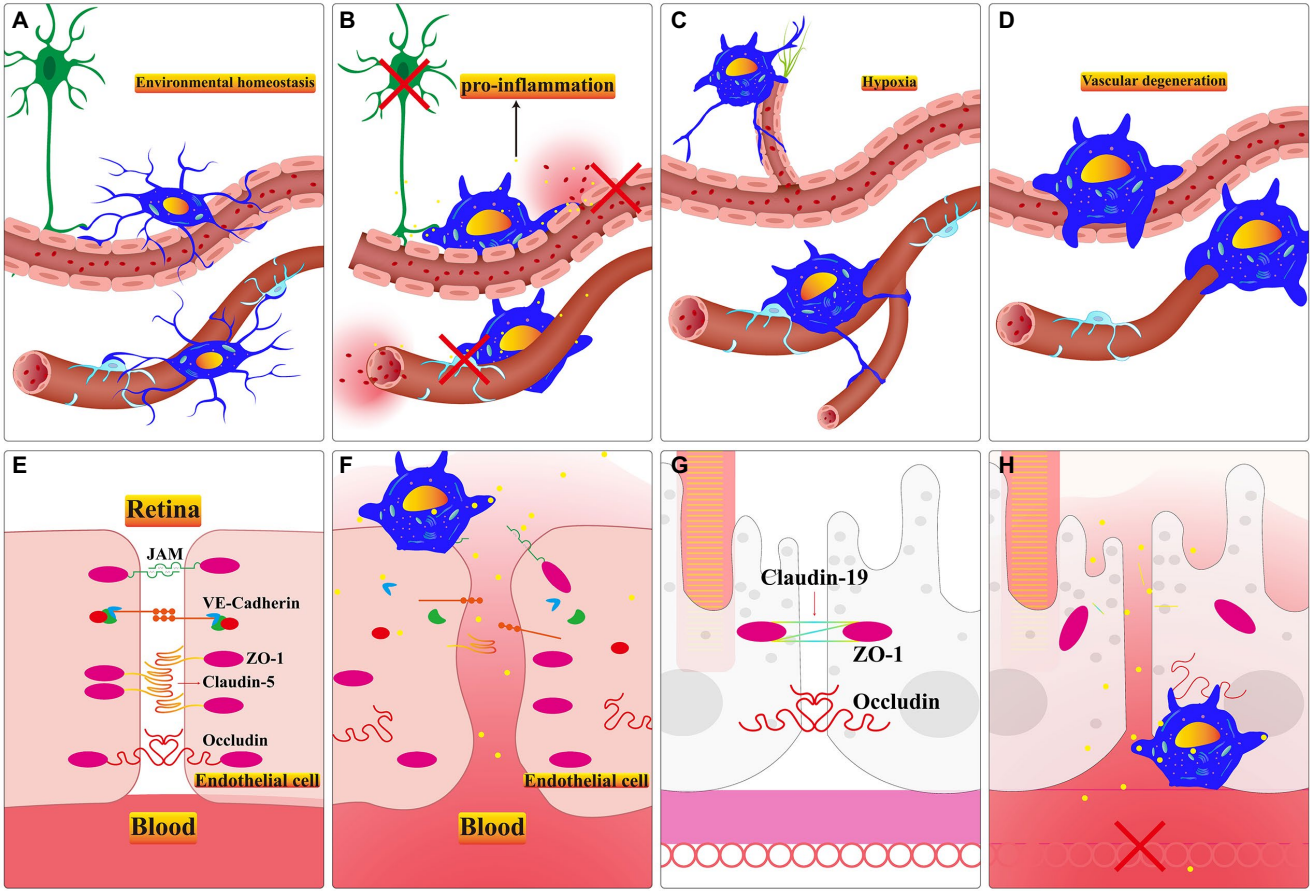
**(A)** Microglia monitor the retinal pericytes and endothelial cells and regulate the rate of retinal blood flow in healthy retina. B-D. The microglial responses in different retinal dysfunction environments. **(B)** Activated microglia promote apoptosis of neuronal cells, endothelial cells, and pericytes. Persistent activation of microglia alters the permeability of endothelial cells and promotes the appearance of vascular leakage. **(C)** Microglia guide the formation of neovascularization when the retina stays in a comparatively hypoxic environment. **(D)** In an inflammatory environment, activated microglia phagocytose the neovascular plexus or engulf apoptotic endothelial cells. **(E)** The major types of tight juctional molecules between endothelial cells of the iBRB in healthy retina. **(F)** Inflammatory factors secreted by activated microglia facilitate the downregulation of tight junctional molecules between endothelial cells, and vascular leakage occurs. **(G)** The significant types of tight junctional molecules between RPE cells of the oBRB in healthy retina. **(H)** Inflammatory factors secreted by activated microglia accelerate the downregulation of tight junctional molecules between RPE cells, capillary leakage, and RPE cells apoptosis. Choroidal neovascularization invasion to subretinal space in a relatively hypoxic environment.

Microglia settle in the retina through the optic nerve, vitreous body, and ciliary body before retinal vasculature matures (Diaz-Araya et al., 1995). Their ramifications contact endothelial stalk cells and filopodia on tip cells in the period of mouse retinal vasculature development (Checchin et al., 2006). And microglia may lead tip cells to clarify the direction of neovascularization which is ultimately limited in retinal GCL, IPL and OPL layers (Haupt et al., 2019). Sharing resemblances with the brain, after treatment with lipopolysaccharide, the primary microglia up-regulate the *VEGF-A* and *PDGF-BB* expression level of RMEC which promotes the ability of tubes formation, migration and proliferation (Li et al., 2014; Ding et al., 2018). After using PLX5622 to deplete microglia, retinal choroidal vessels begin to atrophy, and RPE present dysfunction (Yang et al., 2020). Microglia and retinal vessels are positively correlated in terms of quantity during development (Checchin et al., 2006; Zeng et al., 2022). The diminished number of retinal microglia result in sparse vascular density during the second and fourth postnatal days in *CSF*^−/−^ deficient mice (Rymo et al., 2011). Exogenous microglia within intravitreal injection promotes the decreasing density and area of vascular triggered by resident microglia depletion during the retina development process (Checchin et al., 2006). Activated pro-inflammatory microglia are involved in the remodeling of retinal vasculature in the PKD model (Chen et al., 2021). This evidence is sufficient to demonstrate the indispensable role of microglia in adjacent retinal vasculature (Figures 3A–D).

#### 2.2.5. Microglial relationship with BRB

Altered endothelial cell permeability is the central mechanism of BRB dysfunction during retinopathy (Sun et al., 2021). The defective BRB accelerates the death of the cone leading to swift vision degeneration (Ivanova et al., 2019). Targeting activated microglia is a crucial factor in repairing damaged BRB. They recruit neutrophil infiltration, aggravating the damage to the blood-retina barrier and optic nerve cell death in the retinal vein occlusion model (Jovanovic et al., 2020). Microglia can be activated by hyperglycemia in the retinal environment (Zhang et al., 2019). And the activated microglia engulf endothelial cells which exacerbates the phenomenon of acellular capillaries and albumin leakage in the diabetic model (Xie et al., 2021). Inflammatory factors secreted by activated microglia aggravate the damage of BRB (Usui-Ouchi et al., 2020). Pro-inflammatory microglia activate the *TLR4/MyD88/NF-κB p65* signaling axis and the *NF-κB p65* nuclear translocation promotes the inflammatory factors resulting in the BRB breakdown (Fang et al., 2021). In the co-culture system, unstimulated microglia promote the expression of endothelial tight junction protein (TJP), Zonula Ocluden-1 (*ZO-1*) and *Occludi*n (Mehrabadi et al., 2017). However, activated microglia secrete IL-1β which stimulates VEGF releasing; subsequently, *VEGF* can down-regulate the expression of *ZO-1* and *Claudin-5* in endothelial cells in hypoxia conditions (Inada et al., 2021). And the ablation of activated microglia alleviates innate immunity stimulated by LPS and protects retinal BRB integrity through up-regulating *Occludin*, *ZO1* and *Claudin-5* (Kokona et al., 2018; Figures 3E–H).

In conclusion, activated microglia produce inflammatory factors and participate in the damage of BRB. In the hypoxia condition, microglia assist neovascularization and guide them incorrect positioning which causes the secondary lesions and accelerates the disorder progression in patients. Therefore, profoundly investigating the characteristics of microglia under pathological conditions may become a key to treating retinal diseases. After this, we choose one system and two targets we are more interested in. They contribute to retinal vasoconstriction, neovascularization and microglial cytoactive.

## 3. Potential targets of microglia in retinopathy

### 3.1. The microglial renin-angiotensin system contributes to retinal vasoconstriction and angiogenesis

Renin-angiotensin system was initially considered a humoral system that governed blood pressure and water-sodium homeostasis. In recent studies, researchers find that renin-angiotensin system has the function of regulating ocular circulation and balancing intraocular pressure. The upregulation of the renin-angiotensin system contributes to the formation of blood vessels in the developing retina (Sarlos and Wilkinson-Berka, 2005). Dysregulation of the renin-angiotensin system is commonly seen in retinopathy, such as ROP. Blocking the renin-angiotensin system could postpone neovascularization forming (Misrak Tadesse et al., 2001).

In the renin-angiotensin system, renin, a rate-limiting enzyme which the precursor is pro-renin, disassembles angiotensinogen (*Agt*) encoded by the AGT gene to angiotensin I (*Ang I*). *Ang I* subsequently hydrolyze to effector molecule angiotensin II (*Ang II*) under angiotensin-converting enzyme (*ACE*). In the model of proliferative Diabetic Retinopathy (DR) and Age-Related Macular Degeneration (AMD), *Ang II* upregulates the secretion of angiogenic factors and growth factors, provokes microglial activation and leads to hypertension (Moravski et al., 2000; Shi et al., 2010; Hatzopoulos et al., 2014). And up-regulating the expression of retinal Ang II promotes the expression of TNF-α secreted by the activated glial cells which contributes to the death of RGC (Jeon et al., 2022). Activated microglia secrete Agt and Ang II resulting in angiogenesis during tissue injury or disease (Mills et al., 2021).

**Figure 4:**
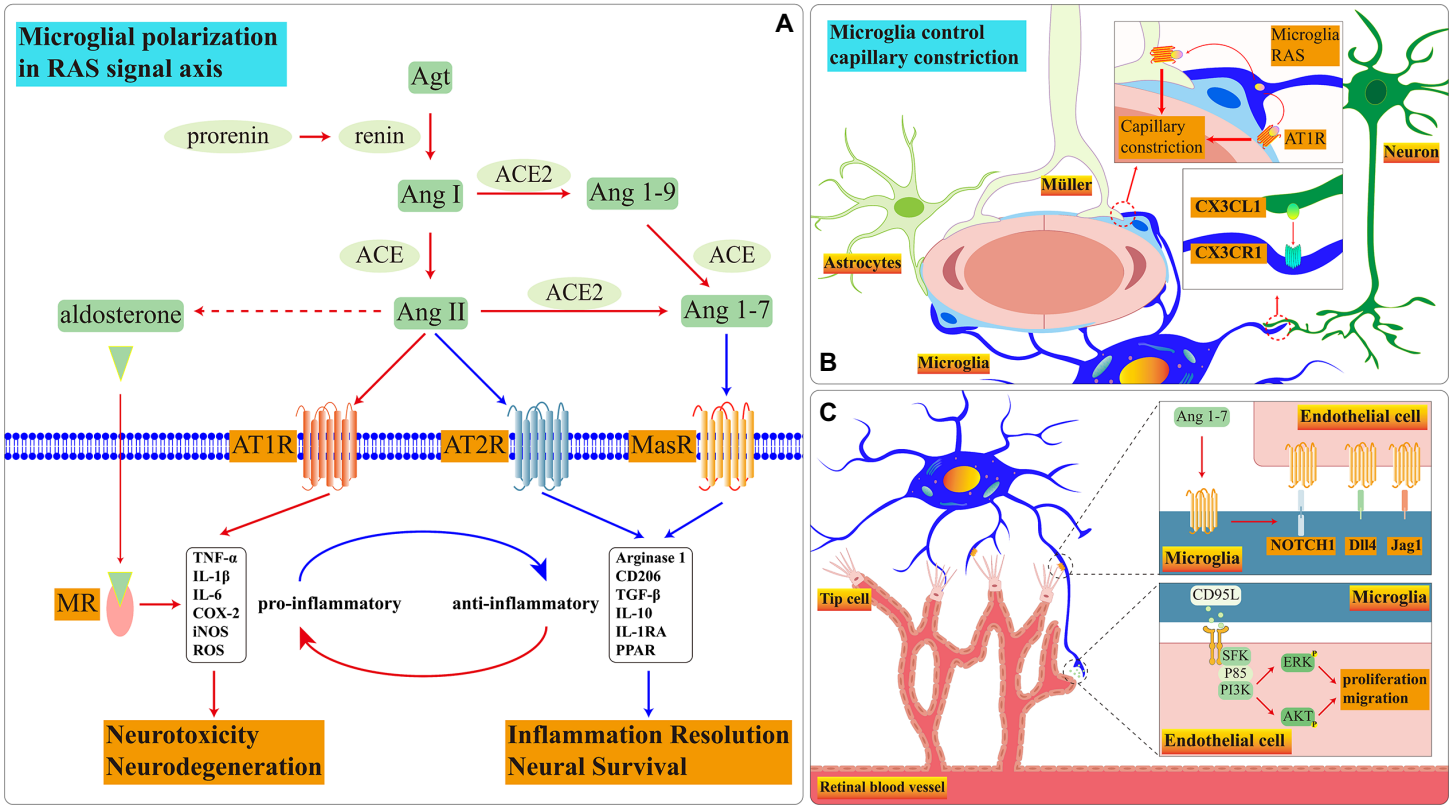
Schematic diagram of RAS signal pathways in microglia. **(A)** During disease onset, the RAS in microglia is imbalanced. Ang II is biased towards binding to *AT1R* and *MR* which promote microglia to pro-inflammatory functions. Among these receptors, the presence and function of *AT2R* in retinal microglia remain to be demonstrated. **(B)** The crosstalk among three glia cells controls blood flow rate through RAS. **(C)** Microglial *MasR* controls blood vessel formation during retinal development.

However, the functions are dissimilar when Ang II binds with various receptors. The common receptors of *Ang II* are angiotensin subtype-1 receptor (*AT1R*), Ang II type 2 (*AT2R*) and *MAS-R* (Figure 4A).

#### 3.1.1. Renin-angiotensin-aldosterone (RAAS) and AT1R

The human retina has locally subsisting RAAS systems (Wagner et al., 1996). RAAS controls vasoconstriction, regulates the release of vascular endothelial growth factor (*VEGF*) to participate in angiogenesis and increase vascular permeability. Moreover, *AT1R*, as an essential receptor with cardiovascular homeostasis, facilitates the efficacy of Ang II. And Ang II participates in aldosterone release under the action of aldosterone synthase (Maning et al., 2017). Aldosterone further binds to mineralocorticoid receptor (*MR*) regulating internal water-electrolyte balance and influencing cardiovascular diseases (Waanders et al., 2011). In addition, the aldosterone with the intravitreal injection can mimic clinical symptoms of central serous chorioretinopathy (CSC; Yu et al., 2022).

Retinal microglia have RAAS. In healthy conditions, Microglial *CX3CR1* receives neuronal *CX3CL1* signal which may govern the velocity of retina blood flow through RAAS (Mills et al., 2021; Figure 4B). When the exogenous Ang II binds to *AT1R* on the surface of N9(a microglia cell line) and primary microglia, the *ROCK* activation stimulates the NOX activation *via P38* (Rodriguez-Perez et al., 2015). *NOX* is one of *ROS* manufacturers which facilitates the process of retinopathy (Ahmad et al., 2021). In addition, the binding of *Ang II* to *AT1R* motivates the translocation process of *NF-кβ* and *STAT3* which accelerates the *TNF-α* releasing (Abadir et al., 2011). Adding *AT1R* antagonist effectively reverses microglial inflammatory phenotype and reduces the release of inflammatory factors stimulated by exogenous *Ang II* (Phipps et al., 2018). *AT1R* up-regulates at 12 h after ischemia, the candesartan, as an *AT1R* antagonist, effectively reduces the inflammation (Fukuda et al., 2010). The *AT1R* antagonist diminishes *NF-Kβ* nuclear translocation and STAT3 phosphorylation which declines the release of *TNF-α, IL-10, ROS* and nitrite accumulation in BV2 microglia treated with LPS (Bhat et al., 2016).

Microglia also express MR and aldosterone synthase. Microglial density significantly up-regulates in both REN-2 transgenic rats (rats overexpress renin and Ang II) and the Oxygen-Induced Retinopathy(OIR) model; Valsartan (an antagonist of *AT1R*) and Spironolactone (an antagonist of *MR*) effectively diminish the *VEGF, CCL5* and *IFN-γ* secreted by the primary microglia *in vitro* hypoxia condition (Rana et al., 2020). Activated microglia can promote retinal neovascularization in some cases (Hatzopoulos et al., 2014). Using FAD286 (an aldosterone synthase inhibitor) reduces the density of *Iba1^+^* microglia and inhibits approximately 89% of neovascularization and 67% of neovascular tufts in the OIR model (Deliyanti et al., 2012).

According to the clues above, we can summarize microglia may regulate the velocity of retina blood flow through RAAS in healthy conditions. However, in chronic diseases, RAAS converts microglia to the pro-inflammatory phenotype, which releases inflammatory factors and injures the integrity of BRB. Reactive microglia promote angiogenesis when the survival environment situates in relative hypoxia.

#### 3.1.2. Ace/Ang II/AT2R

The *ACE/Ang II/AT2R* signaling pathway is associated with anti-angiogenesis, anti-inflammatory and partly balances the influence of RAAS (Tao et al., 2016; Carey, 2017). The expression of *AT2R* decreases with age and is lower than AT1R in the adult mouse retinas (Yoon et al., 2016; Verma et al., 2019). The up-regulation of *AT2R* effectively prevents damage to the optic nerve and the polymorphism of G/A at the 1,675 site may be related to arteriolar diameter (Kurihara et al., 2006; Liu et al., 2011). Moreover, the activation of the *AngII-AT2R* signaling pathway can reverse the microglial inflammatory phenotype (Gao W. et al., 2022).

Utilizing exogenous *Ang II* activates the *GSK3β* through upregulating the phosphorylation of Y216 and downregulating the phosphorylation of S9 which degrade the *NRF2* expression and inhibit microglial antioxidant capacity; the escalation of Ang II concurrently leads to mitochondrial dysfunction through stimulating phosphorylation of *PKCα/β* and *P-PKCδ*, activating phosphorylation of NOX-2^p47phox^ and generating *ROS* accumulation (Bhat et al., 2019). *AT2R* binding to *AngII* stimulates the activation of *PP2A* (Guimond and Gallo-Payet, 2012). *PP2A* guarantees neuronal survival and silences *PKC/ERK/NF-кB* signaling pathway to prevent inflammatory cascade reaction stimulated by LPS (Egger et al., 2003; Bononi et al., 2011). CGP42112A, as an *AT2R* agonist, induces *PP2A* activation by declining the *p-Y307-PP2A* expression and inhibits *NOX-2* activation by declining the *P-S345-P47^phox^* expression; in addition, CGP42112A reduces the ROS production induced by Ang II and converts microglial phenotype from pro-inflammatory to anti-inflammatory (Bhat et al., 2019). Delaying administration of C21 (an agonist of *AT2R*) after 3 days post-stroke aggrandizes the number of anti-inflammatory microglia and effectively improves the rate of survivability, sensorimotor and cognitive deficits (Jackson et al., 2020; Sumners et al., 2020). Adding CGP42112 boosts the proliferation ability of microglial; the newborn cells predominantly present the ramified structure in peripheral infarct and the area of the infarct is diminished after 3 days of stoke; however, using PD4123319 (an *AT2R* antagonist) reverses the protective function of CGP42112 (McCarthy et al., 2012). We speculate that CGP42112 may promote anti-inflammatory microglia which maintain neuronal survivability. And in the central infarct core, the continuous release of inflammatory factors stimulates microglia to form different phenotypes with microglia in the peripheral infarct area. Whereafter, McCarthy et al. find that under the C21 stimulation, microglia releases brain derived neurotrophic factor (*BDNF*) which protects neuron survival and improves vasodilation (McCarthy et al., 2014). In addition, the upregulation of VEGF stimulated by PD123319 promotes the proliferation and migration ability of endothelial cells; on the contrary, CGP42112A selectively inhibits vascularization driven by VEGF in the OIR model (Carbajo-Lozoya et al., 2012).

Therefore, we speculate there may be a similar mechanism to brain-derived microglia in retinal microglia. Retinal microglia may preserve neuronal survival and blood vessels through the *ACE/Ang II/AT2R* signaling pathway which is expected to treat neoangiogenic retinopathy.

#### 3.1.3. ACE2/Ang1-7/MasR

*ACE2/Ang-(1–7)/MasR* promotes vasodilation and has demonstrated anti-inflammatory properties (Liu et al., 2016). ACE2, a homolog of ACE, hydrolyzes *Ang I* and *Ang II* to *Ang1-7*; *Ang1-7* binds to Mas receptor (*MasR*) which is encoded by MAS1 proto-oncogene (Mas1; Jiang et al., 2013). In oxidative stress injury induced by *Ang II*, the *Ang1-7* activation protects the survival of dopaminergic neurons, alleviates microglia-induced inflammatory responses and enhances the survival rate of hypertensive stroke rats model (Regenhardt et al., 2014; Liu et al., 2016; Costa-Besada et al., 2018).

Retinal microglia express *MasR* which gradually decreases with age (Tuhina Prasad and Li, 2014). In primary retinal microglia, the expression of *MasR* is up-regulated following the hypoxia condition which is a necessary element to stimulate angiogenesis in retinal development process (Gariano and Gardner, 2005; Foulquier et al., 2019). In *MAS1* deficient mice model, the rate of angiogenesis is slower than in controls, and the number of perivascular microglia and filopodia of endothelial tip cells is significantly decreased; under the stimulating of *MAS* agonist AVE0991, the mRNA expression level of *IL-10*, *Notch1*, *Dll4*, and *Jag1* are increased (Foulquier et al., 2019). The contact points between microglial ramifications and endothelial cells activate the *Notch1* signal which facilitates tip cells to recruit microglia during retinal development in newborn mice (Outtz et al., 2011). Microglia can stimulate vessel sprouting and branching of aortic ring explants through soluble factors and angiogenic factors; in turn, aortic ring explants recruit microglia to migrate toward them (Rymo et al., 2011). Therefore, we have reasonable doubt to suspect that microglia interact with endothelial cells and guide the tip cells in the neovascular to migrate toward the retinal periphery through the *MAS* signaling pathway during retinal development (Figure 4C).

The primary microglia of the brain also express *MasR* (Rivas-Santisteban et al., 2021). Nevertheless, renin-angiotensin system becomes unbalanced under the long-term stimulation of LPS in the brain; when *ACE2/ Ang(1–7)/MasR* signal axis is stimulated by AVE0991, microglia convert their phenotype from pro-inflammatory to anti-inflammatory (Dang et al., 2021). Utilizing DIZE, an ACE2 activator, diminishes inflammation of activated microglia, up-regulates cell survival proteins and down-regulates neuronal apoptotic proteins through stimulating the *ACE2/ Ang (1–7)/MasR* signal axis in the 6-OHDA induced Parkinson’s model; nevertheless, the A-779, a MasR antagonist, balances the protective affection of DIZE (Gupta et al., 2022). *MasR* can form a dimer with AT1R or AT2R. The expression of these dimers in striatal microglia is higher than those in cerebral neurons; when microglia are treated with IFN-γ and LPS, the expression of *AT1R-MasR* is up-regulated and the expression of *AT2R-MasR* is down-regulated; Rivas et al. propose the possibility of forming heterotrimer among *AT1R, AT2R* and *MasR* (Rivas-Santisteban et al., 2021).

Based on the above clues, we consider the dysfunction of the microglial renin-angiotensin system loses the ability to control retinal vasoconstriction in retinal vascular malformation diseases which reduces the adequate oxygen supply to retinal neurons leading to the deterioration of vision. The molecular relationship among AT1R, AT2R, and MasR needs to be further verified in retinal microglia (Figure 4).

### 3.2. Microglial CD39 may provide anti-inflammatory treatment and protect BBB integrity during retinopathy

In the mammalian retina, ATP and adenosine (*ADO*) are crucial molecules that participate in vascular remodeling and form the neurovascular coupling (Zeiner et al., 2019; Losenkova et al., 2022). Abnormally elevated expression of ATP is a common phenomenon in many retinopathies. There are an abundant supply of adenosine triphosphate (*ATP*) ecto-nucleotidases near retinal blood vessels which are classified into three main types: nucleoside triphosphate diphosphohydrolase 1 (*NTPDase1*/*CD39*), *NTPDase2* and ecto-5′-nucleotidase (*CD73*). Thereinto, *CD39* is an extracellular enzyme encoded by *ENTPD1* which hydrolyzes ATP to adenosine diphosphate (*ADP*) and adenosine (*ADO*) by cooperating with *CD73*. And retinal *CD39* are mainly produced by microglia and endothelial cells. At the early stage of retinopathy, the *CD39* expression is significantly up-regulated to maintain the balance between *ATP* and *ADO*; when retinal disease develops in the advanced stage, the number of *CD39^+^* microglia decrease prominently and the balance between *ATP* and *ADO* is broken; The overladen ATP in the retinal microenvironment mediate inflammatory reaction that leads to neuronal death and M1 microglial activation (Lu et al., 2015; Hu et al., 2017; Rodrigues-Neves et al., 2018).

In addition to eliminating the redundant *ATP* in the internal environment, *CD39* can remodel the anti-inflammatory properties of macrophages (Villacampa et al., 2015; Jakovljevic et al., 2019). *CD39* and *CD73* synergistically inhibit the multiplication capacity of T cells and decrease the production of inflammatory factors in the EAU mouse model (Chen et al., 2016). Similarly, in type I diabetic hypothalamus, the expression of *CD39* in microglia and blood vessels is diminished by 30%, and the integrity of the blood–brain barrier is impaired; Minocycline can effectively promote the expression of *CD39* and the pro-inflammatory microglial activation is decreased (Bulavina et al., 2013; Hu et al., 2015).

**Figure 5:**
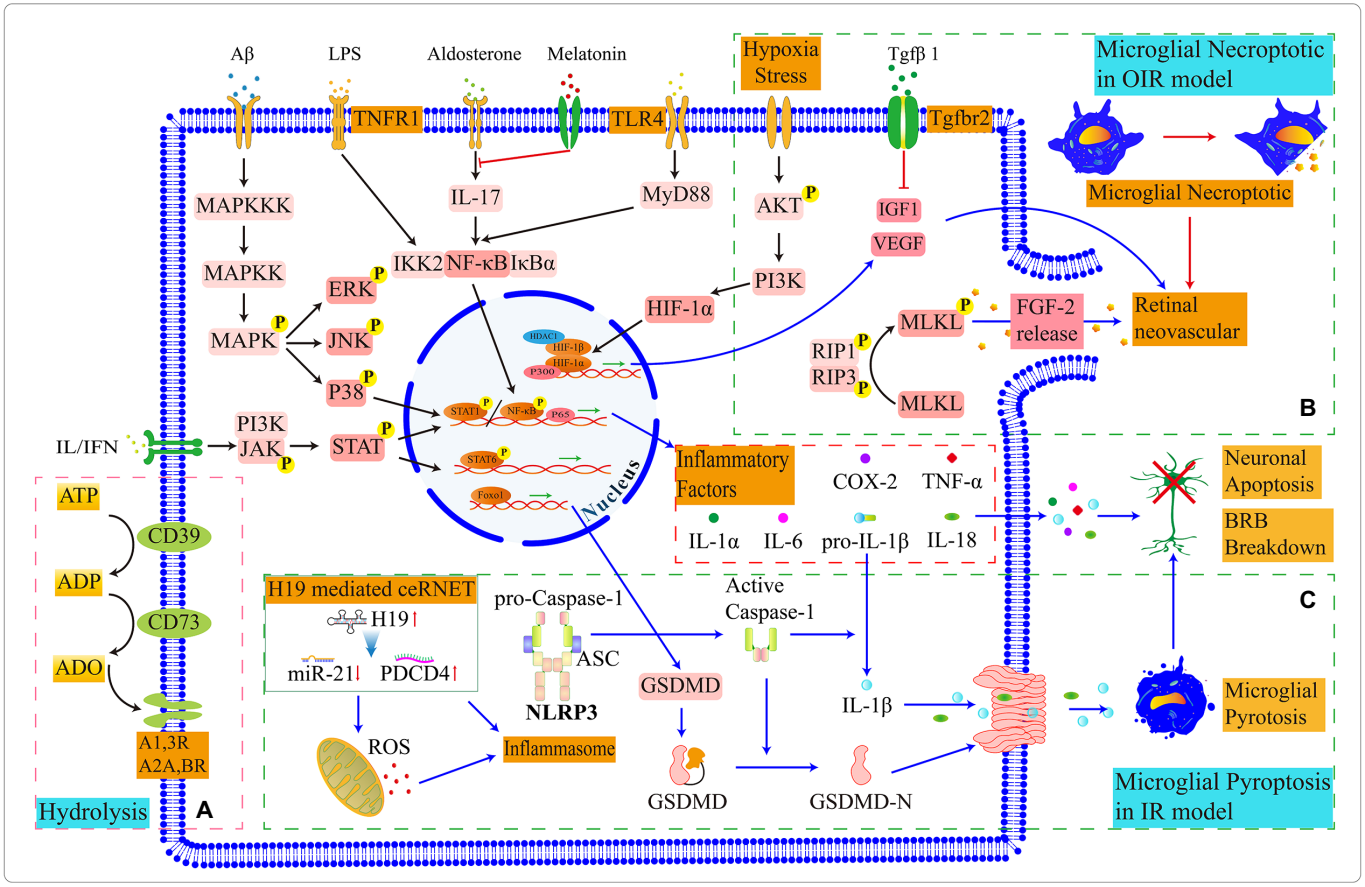
Schematic diagram of partly signal pathways in retinal microglia. **(A)** Microglia express *CD39* and *CD73* that progressively hydrolyze *ATP* to *ADO* together. **(B)** In the OIR model, microglial necrosis promotes the leakage of FGF, which stimulates retinal neoangiogenesis. Microglia express proangiogenic factors to promote retinal vascular proliferation, and the binding of **(C)** In the IR model, a competing endogenous RNA network (ceRNET) stimulates microglial pyroptosis. The maturation of pro-caspase1 in inflammasome and the activation and assembly of *GSDMD* protiens lead to the leak age of inflammatory factors, which ultimately result in the apoptosis of retinal neurons and damage to the BRB. The switching status of microglial signal pathways in different retinal environments and the target genes in upstream and downstream need to be further explored.

The *ADO*, the hydrolysate of *ATP*, participates in the microglial contracting branches process and recruits them to migrate toward the lesion area (Matyash et al., 2017; Lim et al., 2018). The microglia migration capacity decreases significantly in *CD39*-deficient mice; after adding exogenous ecto-nucleotidases or *ADO*, microglial migration ability recovered (Farber et al., 2008).Microglial *CD39* mitigates neuronal overactivation through the *ATP/AMP/ADO/A1R* signaling axis (Badimon et al., 2020). *ADO* can bind to different receptors and perform different functions. Adenosine A1 receptor (*A1R*) and *A2aR* are involved in regulating angiogenesis, blood flow rate and inflammatory response. For example, in the diabetic retina, the *ADO* releasing and formation stimulated by Triamcinolone promotes the *A1R* activation which prevents cells from osmotic swelling and results in ion efflux through potassium and chloride channels (Wurm et al., 2008). And the *A2aR* activation can inhibit phagocytic activity and migration capacity of primary microglia (Ingwersen et al., 2016). The downstream response mechanism of retinal microglia receiving *ADO* signals is still unclear and needs further exploration (Figure 5A).

Interestingly, microglia create a spatially arranged network in the retinal parenchyma and form a local “purinergic junctions” system with *CD39^low^/CD73^−^* neuronal cell bodies and *CD39^high^/CD73^−^* retinal blood vessels through their *CD39^high^/CD73^low^* branches (Losenkova et al., 2022). Further exploring the mechanisms through which a broad spectrum of soluble and membrane-binding enzymes synergistically regulates purine levels in the retina may serve as potential therapeutic targets for the treatment of retinopathy (Zeiner et al., 2019). Increasing the number of CD39^+^ microglia subset may contribute to the restoration of the retinal barrier and alleviate inflammation and reduce retinal vascularization.

### 3.3. Microglial Tgfβ1 and its receptor TβRII involve in vascular remodeling and cellular phenotype conversion

Transforming growth factor beta (*Tgfβ*), as a multifunctional cytokine which is associated with AMD susceptibility, is vital to sustain the specificity of microglia, ensure the stability of retinal vascular endothelial cells and BBB (Walshe et al., 2009; Braunger et al., 2013; Bohlen et al., 2017; Zarkada et al., 2021). *TGFβ* can up-regulate *SNAIL* through AKT and polarize macrophages to anti-inflammatory phenotype; when blocking *TGFβ/SNAIL* signal transduction, they promote the output of inflammatory factors and convert their phenotype to pro-inflammatory (Zhang et al., 2016). The *Tgfβ* up-regulation inhibits pro-inflammatory microglial activation and reduces the *TNF-α* and *IL-6* releasing in microglia during retinopathy (Kim et al., 2004; Taylor et al., 2017; Yang et al., 2021).

In mammals, three members of the *Tgfβ* isoform family encoded by independent genes are identified: *Tgfβ1, Tgfβ2* and *Tgfβ3* (Anderson et al., 1995). *Tgfβ1*, as a potential therapeutic target, reduces microglia-mediated neuroinflammation and improves outcomes of intracerebral hemorrhage after acute injury (Taylor et al., 2017). Although the expression of *Tgfβ1* is the lowest one of the *Tgfβ* family in the adult retina, the ability of *Tgfβ1* has been proven to induce microglial conversion from the pro-inflammatory phenotype to anti-inflammatory phenotype which is related to neuroprotection and the anti-inflammatory treatment (Tosi et al., 2018; Caruso et al., 2020). In Rho^−/−^ and PDE6β^mut/mut^ mouse model of retinitis pigmentosa, AAV8-mediated supplementation of *Tgfβ1* effectively prolongs the cone degeneration time through up-regulating *Spp1* and down-regulating *Gas6* of microglia, thereby indicating the therapeutic potential of precisely polarizing pro-inflammatory to anti-inflammatory microglia (Wang et al., 2020). And *Tgfβ1* can also promote the up-regulation of CD73, inhibit the proliferation of activated T cells and reduce inflammation of the internal environment in the EAU model (Chen et al., 2016). Surprisingly, *Tgfβ1* expressed by microglia frequently associates with vascular remodeling. In the microglial autocrine *Tgfβ1* signaling pathway, *Kindlin3*, as an intracellular adapter molecule, is associated with microglial polarization; when *Kindlin3* is knockout, high myosin contractility contributes to *ERK* phosphorylation and further promotes the overexpression of *Tgfβ1* which results in angiogenesis (Dudiki et al., 2020).

The *Tgfβ* receptor consists of two structurally similar sub-families (*TβRI* and *TβRII*) and a transmembrane proteoglycan (*beta-glycan*/*TβRIII*). Thereinto, *TβRII* belongs to the serine and threonine transferase receptor, which is highly expressed in retinal microglia and endothelial cells (Ma et al., 2019). After *Tgfβ* ligation to *TβRII*, *SMAD2* and *SMAD3* are phosphorylated and form complexes with *SMAD4* which transfer to the nucleus and further promote the transcription of target genes (Javelaud and Mauviel, 2004). In *TβRII*–deficient condition, the retina presents a critically pathological change in structure and function, such as the shortage of pericyte differentiation and retinal capillaries, which leads to microaneurysms, hemorrhages, microglial activation and proliferative retinopathy (Braunger et al., 2015). According to the collaborative genome-wide association study, *TβRII*, as a receptor for *Tgfβ* signaling pathway, was also associated with AMD susceptibility (Fritsche et al., 2013).

Nevertheless, because of the postnatal lethality of *TGFβ1*-deficient mice or lethal embryonic phenotypes of *TβRII-deficient* mice, was limited to investigate how *TGFβ1* regulates microglial activation through *TβRII* in adults (Shull et al., 1992; Oshima et al., 1996). Therefore, Zoller et al. constructed *Cx3cr1^CreERT2^:Tgfbr2^fl/fl^* mouse model to persuade the conditional deletion of TβRII in adult microglia and found that *TβRII-deficient* microglia change the original morphology, up-regulate microglial activation and increase phosphorylation of *TAK1* (Zoller et al., 2018). The ablation of *TβRII* in retinal microglia induces the secondary apoptosis of Müller and reduces mRNA expression level of microglial ‘sensome’ transcripts, such as *Siglech* (Hickman et al., 2013; Ma et al., 2019). Although *TβRII*-deficient microglia do not induce obvious changes in the retinal structure of adults, they promote pathological angiogenesis after laser injury (Ma et al., 2019). Similarly, in OIR, *TβRII*-deficient microglia secrete chemokines and up-regulate *Igf1* expression which exacerbates retinal neovascularization plexus forming (Usui-Ouchi et al., 2022; Figure 5B).

According to these shreds of evidence, we draw partly microglial signaling pathways we are more interested in (Figure 5). Then we speculate that retinal *TGFβ1* promotes the up-regulation of *CD73*; *CD39* in microglia or endothelial cells cooperates with *CD73* to hydrolyze *ATP* to *ADP* and *ADO*; and the hydrolysis production *ADO* can recruit microglia. In retinal vascular diseases, such as diabetic retinopathy and retinopathy of prematurity, the expression of *ATP* and *ADO* are upregulated. This may partly explain why microglia appear in adjacent vessel and injury sites. Intracellular molecular mechanisms in microglia responding to *ADO* need to be explored in depth. In addition, the *Tgfβ1/TβRII* signaling pathway in microglia effectively decreases microglial activation and exhibits a protective effect on neurons in retinopathy. And the disruption of the *Tgfβ1/TβRII* signaling pathway in microglia accelerates pathological angiogenesis during retinal injury and hypoxic processing. The relevance of *Tgfβ1* to microglia *CD39* and the renin-angiotensin system is currently unknown.

## 4. Effect of microglia on retinal vasculature and BRB in various retinopathy models

### 4.1. Microglia in diabetic retinopathy

DR, characterized by the apoptosis of pericytes and endothelial cells as an early clinical feature, results in the damage of BRB integrity and leakage of plasma. Although microglia may be a member of the BRB and act as a secondary protective barrier for extravasated proteins, we cannot ignore the inflammatory response induced by activated microglia. Microglia is the main source of retinal *TNF-α* after retinal injury. At 3 h of *TNF-α* intravitreal injections, endothelial cells appeared necrotic and revascularization began at 24 h injection (Claudio et al., 1994). The massive *IFN-γ* and *IL-6* in DR activate microglial STAT3 phosphorylation which stimulates TNF-α secretion, suppresses the kinase activity in *AKT/p70S6* signal axis and leads to the apoptosis of pericytes. Under *IL-6* stimulation, the activated microglia recruit to the vicinity of RPE and secrete *TNF-α* to downregulate the expression of *ZO-1* and Occludin in the RPE which disrupt the integrity of oBRB; whereas using *STAT3* inhibitors modify the influence of microglia to RPE (Jo et al., 2019). With a similar mechanism, the activated microglia increase the permeability of endothelial cells in iBRB (Yun et al., 2017).

In addition, the normal retina responds to light stimulation through neurovascular coupling which expands the blood flow per unit time; whereas in the early stages of type I and II diabetes mellitus, patients normally present a deferred reaction under the stimulation of flickering (Lim et al., 2014). Microglial branches contact the pericytes, endothelial cells, and neuronal synapses in the retinal vasculature and regulate retinal vasoconstriction through the renin-angiotensin system; nevertheless, microglia lost this ability in the early STZ-induced mouse model (Mills et al., 2021). Asiatic acid upregulates the protein arginine (*Arg-1*) expression and reduces *NF-κB p65* nuclear translocation in microglia; the microglial phenotype is reversed and alleviates the disease process of DR (Fang et al., 2021). Erythropoietin protects the BRB by increasing phosphorylation of the *Src/Akt/cofilin* signaling axis to inhibit microglia engulfing endothelial cells (Xie et al., 2021). CD5-2, as a novel oligonucleotide-based drug, enlarges the VE-cadherin transcription which is silenced by *miR-27a* in endothelial cells and defends the integrity of BRB and preserves the coverage of pericytes; the activation state of microglia is suppressed after treatment with CD5-2 (Ting et al., 2019). Melatonin can inhibit microglia activation through the *PI3K/Akt/Stat3/NF-κB* signal axis and protect pericytes from apoptosis (Tang L. et al., 2022). And *MicroRNA-93-5p* can restrain M1 microglia activation by silencing *STAT3* (Wang et al., 2021).

### 4.2. Microglia in age-related macular degeneration

AMD is a neurodegenerative disease of retinal macular degeneration which is associated with age and can mainly divide into three types: (1) Geographic atrophy; (2) choroidal neovascularization; (3) retinal angiomatous proliferation (RAP). In healthy human retinas, the macular region has no blood vessels on account of the abundantly antiangiogenic factors expressed by cells (Lutty and McLeod, 2018). And the high density of cones in this area indicates more susceptibility to oxidative stress.

Quite unlike humans, the retina of mice does not have macular structures. Hence, external assistance is needed to establish disease models and explore the mechanisms involved in macular degeneration. For example, the NRL-deficient transgenic model assists us in researching retinal degenerative diseases related to cones and decreases the interference of rods (Samardzija et al., 2014). Barben et al., based on this model and knocked out the von Hippel Lindau protein to mimic the degenerative death mechanism of cone under chronic hypoxic conditions in AMD and found that microglia infiltrate to the fundus and the upregulation of *H1F-1A*, *VEGF*, and *Fgf2* related to neovascularization during early retinal development (Barben et al., 2018). In contrast, the sterile 1% NaIO3 can mimic geographic atrophy by injecting the tail vein (Enzbrenner et al., 2021). And in the 5XFAD mouse model, the presence of deposits on the fundus can mimic types 2 and 3 AMD while using the low-dose efavirenz reduces microglial activation, neovascular formation, and accumulations of amyloid β plaques in focus (El-Darzi et al., 2022).

The microglial number in perivascular and subretinal increases with age. The increased number of microglia and lipofuscin deposition synergistically contribute to the risk of the AMD process (Xu et al., 2008). And the structurally altered *HTRA1* contributes to the *Tgfβ* signaling in autocrine microglia which downregulates phosphorylation of *SMAD2* and similarly increases the risk of AMD (Friedrich et al., 2015). Moreover, the high-fat diet activates the immune response of retinal microglia; *IL-1β* released by the activated microglia provokes the cellular iron sequestration reaction which sparks the toxic accumulation of iron in RPE cells; subsequently, the RPE presents oxidative stress and electrophysiological dysfunction facilitating the process of AMD (Sterling et al., 2022).

### 4.3. Microglia in other types of retinal diseases

Autoimmune uveitis (Au) is a chronic inflammatory intraocular disease mediated by the autoimmune system. In AU, the abnormal activation of microglia increase the production of inducible nitric oxide synthase (*iNOS*) which accelerates retinal degeneration and the loss of iBRB integrity, the leakage and abnormal proliferation of capillaries. In the EAU mouse model, the addition of Icariin upregulates *PRDX3* which transfers the microglia phenotype from pro-inflammatory to anti-inflammatory and alleviates the state of illness (Wang et al., 2022). Thereinto, *PRDX3*, a primary isoform of six peroxidases in mitochondria, swiftly scavenges abnormal accumulation of *H2O2* to decrease apoptosis and damage caused by oxidative stress (Rebelo et al., 2021).

In addition, CSC is characterized by the dilation and leakage of choroidal vasculature leading to the accumulation of subretinal fluid and serous detachment of the neurosensory retina which can mimic by using aldosterone with the intravitreal injection (Zhao et al., 2012; Yu et al., 2022). CSC may be associated with inappropriate activation of *MR* (Zhao et al., 2017). The melatonin through intraperitoneal injection rescues microglial infiltration mediated by activation of the *IL-17A/NF-kb* signaling pathway and significantly reduces *CX3CR1* and cyclooxygenase 2 secreted by the activated microglia in the CSC mouse model (Yu et al., 2022).

### 4.4. Microglia in oxygen-induced retinopathy (OIR)

Microglia are involved in neoangiogenesis and are activated prior to neovascularization in the central avascular region during OIR (Ritter et al., 2006; Fischer et al., 2011). At the beginning of the OIR model construction, mice are exposed to an oxygen-rich environment and the capillaries appear to atrophy; when the survival environment returns to the normoxia, neovascularization occurs in the retina. At postnatal 12 days, the *NF-κβ/STAT3* signaling pathway is activated in pro-inflammatory microglia which are presented in the central and peripheral neovascular plexus of the retina; and at postnatal 17 days, anti-inflammatory microglia gradually emerge (Li et al., 2021). During this period, the decreased number of microglia can exacerbate retinal vascular degeneration (Liu J. et al., 2022). Microglia activate the *RIP1/ RIP3* signaling pathway to promote the phosphorylation of MLKL which translocate to the cytoplasmic membrane and regulates the activation of ion channels leading to necroptotic of microglia; the necrotic microglia release FGF2 and HIF-1A which stimulate retinal neoangiogenesis; and the knockdown of RIP3 effectively alleviate angiogenesis (Kubota et al., 2009).

The deletion of *CCN1*, a gene that encodes the extracellular matrix-associated integrin-binding protein, exacerbates the pro-inflammatory responses of microglia and leads to the malformation of retinal vasculature in the OIR model (Yan et al., 2015). And the administration of Celastrol reverses the activation of the *miR-17-5p/HIF-1α/VEGF* signaling pathway which reduces retinal neovascularization by inhibiting the microglial activation and inflammation; the proliferation, migration and tube formation ability of human retinal microvascular endothelial cells are also inhibited *in vitro* culture (Zhao et al., 2022). In addition, KC7F2, a novel molecular compound, reduces retinal angiogenesis by reducing the co-localization ratio between microglia and neovascularization and inhibits the activation of the *HIF1α/VEGF* signaling pathway in human umbilical vein endothelial cells (Tang X. et al., 2022).

### 4.5. Microglia in ischemia–reperfusion (IR) model

The retinal IR model is another common disease model used in experiments to mimic glaucoma, DR and retinal arterial obstruction (Osborne et al., 2004). The activation of immune cells and cytokines can mediate the onset of inflammation and tissue damage; thereinto, microglia are also activated in the early injury process (Zhang et al., 2005). The permeability is quickly increased in vascular endothelial cells accompanied by a strong sterile inflammatory response leading to the BRB breakdown (Muthusamy et al., 2014). On the 13th day of modeling, the average thickness of the GCL and IPL layers decreases by 36 and 5% is reduced in OPL layers; on the 28th day, the overall retinal thickness diminishes by about 10% compared with the control; in addition, after 2 days of IR, the up-regulation of *Occludin pSer490* stimulates the *Occludin* degradation through ubiquitination predicting that TJPs initiate hydrolysis in retinal endothelial cells and inflammation gradually subsides by the fourth week of IR modeling; the activated microglia engulf optic nerve cells and participate in vascular permeability; the administration of minocycline can improve the iBRB repair and reduce the abnormal activation of microglia in the early stage (Abcouwer et al., 2021).

Long non-coding RNA(lncRNA)-*H19* is a key target of IR-induced inflammation; lncRNA-*H19* reduces the *miR-21* and promotes the *PDCD4* expression in the competing endogenous RNA network which activates inflammasome; subsequently, the activated inflammasome prompt the *caspase-1* maturation and induce the microglial pyroptosis through *GSDMD* protein; Microglial *IL-1β* and *IL18* release to the retina and participates in inflammatory response (Wan et al., 2020; Figure 5C).

The inflammatory response from inappropriately activated microglia can exacerbate the retinopathy and the death of pro-inflammatory microglia at later stages is fatal to the disease. Therefore, transferring pro-inflammatory microglia to anti-inflammatory microglia is feasible (Table 1).

**Table 1:** Potential drugs treat the damaged BRB and the retinal neovascularization.

Drugs	Simulation of disease	Study Model	Pathways	Main outcomes	References
Melatonin	Acute glaucoma	Acute ocular hypertension	*Inflammasome/GSDMD RIP1-RIP3/MLKL*	↓ P-RIP3 And Iba1^+^/ IL-1β^+^ microglia	Ye et al. (2022)
				↓ Microglial pyroptosis and inflammation	
				↓ RGC: Caspase-3-dependent apoptosis	
	Central serous chorioretinopathy	Aldosterone	*IL-17A/NF-ΚB*	↑ Tight junction protein in BRB	Yu et al. (2022)
				↓ Microglial infiltration	
				↓ Matrix metalloproteinases	
				↓ Inflammatory factors	
	Diabetic retinopathy	STZ	*PI3K/Akt/Stat3/NF-ΚB*	↑ Anti-inflammatory microglial	Tang et al. (2022)
				↑ BRB function	
				↓ Pericyte loss	
				↓ iBRB leakage	
				↓ Activated microglial number	
	Retinitis pigmentosa	RD10	*None*	↑ The thickness of the ONL	Xu et al. (2017)
				↑ Photoreceptor cells survival	
				↓ Müller cell gliosis	
				↓ Retinal inflammatory response	
	Retinopathy of prematurity	OIR	*HIF-1Α/VEGF*	↑ The formation of tip cells	Xu et al. (2018)
				↓ Vascular leakage	
				↓ Pathological neovascularization	
				↓ Microgliosis	
				↓ Inflammatory factors	
	Age-related macular degeneration	Laser	*RhoA/ROCK*	↓ Choroidal neovascularization	Xu et al. (2020)
				↓ Vascular leakage	
				↓ Vascular proliferation	
Asiatic Acid	Diabetic retinopathy	STZ	*TLR4/Myd88/NF-ΚB P65*	↑ Tight junction protein in BRB	Fang et al. (2021)
				↑ Anti-inflammatory microglia	
Erythropoietin	Diabetic retinopathy	STZ	*Src/Akt/Cofilin*	↑ Endothelial cells survival	Xie et al. (2021)
				↓ Albumin leakage	
				↓ Microglial activation	
				↓ Microglial phagocytosis	
			*None*	↓ Reactive gliosis	McVicar et al. (2011)
				↓ Microglial activation	
				↓ The number of apoptotic cells	
				↓ Acellular capillaries	
	Retinitis pigmentosa	RD10	*None*	↑ Cone cell survival	Sasahara et al. (2008)
				↓ Retinal degeneration	
				↓ Neurovascular degeneration	
	Polycystic kidney disease	PKD	*None*	↑ Retinal thickness	Busch et al. (2014)
				↓ Acellular capillaries	
				↓ Microglial activation	
	Inherited retinal diseases	RCS Rat	*p75^NTR^/pro-NT3*	↑ Ramified microglial infiltration	Shen et al. (2014)
				↑ CD34^+^ cells	
				↓ Retinal capillary dropout	
				↓ Retinal gliosis	
				↓ Focal vascular lesions	
				↓ Reactive gliosis	
				↓ Photoreceptor apoptosis	
CD5-2	Diabetic retinopathy	STZ and OIR	*VE-cadherin/TGFβ and PDGF-β*	↓ Pericyte dropout	Ting et al. (2019)
				↓ Vascular leakage	
				↓ Microglial activation in deep plexus	
Efavirenz	Alzheimer’s disease	5XFAD	*None*	↑ Ramified microglia	El-Darzi et al. (2022)
				↓ Vascular lesion frequency	
				↓ Vascular leakage	
				↓ Focal accumulations of amyloid β plaques	
				↓ Neovascularization	
Icariin	Experimental autoimmune uveitis	IRBP	*GPX4/SLC7A11/ACSL4*	↑ The expression of PRDX3	Wang et al. (2022)
				↑ Anti-inflammatory microglia	
				↓ Retinal inflammation	
	Diabetic retinopathy	STZ	*None*	↑ The basal membrane thickness	Xin et al. (2012)
				↑ Micro-vessel density	
				↑ RGC neurite growth	
Celastrol	Retinopathy of prematurity	OIR	*Mir-17-5p/HIF-1Α/VEGF*	↓ Microglial activation	Zhao et al. (2022)
				↓ Neovascularization	
				↓ Inflammatory cytokine	
	Retinal degeneration	Bright light	*None*	↓ Photoreceptor degeneration	Bian et al. (2016)
				↓ Photoreceptor apoptosis	
				↓ Oxidative stress	
				↓ Inflammatory cytokine	
				↓ Leukostasis	
				↓ Microglial activation	
				↓ Reactive gliosis	
				↓ Proinflammatory genes	
KC7F2	Retinopathy of prematurity	OIR	*HIF1Α/VEGF*	↓ Neovascularization	Tang X. et al. (2022)
				↓ Endothelial cell proliferation	
				↓ Retinal inflammation	
				↓ Leukocytes and microglia	
Progesterone	Inherited retinal diseases	Optic nerve crush	*None*	↑ Anti-inflammatory microglia	Yang et al. (2021)
				↓ Neuronal apoptosis	
				↓ Astrocyte activation	
				↓ Microglial proliferation	
Norgestrel	Retinitis pigmentosa	RD10	*STAT3/GFAP*	↓ Gliosis	Roche et al. (2018)
				↓ Microglial cytokines release	
	Retinitis pigmentosa	RD10	*CX3CL1-CX3CR1*	↑ Neuroprotection	Roche et al. (2017)
				↓ Microglial-derived toxicity	
				↓ Microglial infiltration	
Minocycline	Retinitis pigmentosa	Rho^−/−^	*None*	↑ Photoreceptor survival	Ozaki et al. (2022)
				↓ Disease associated microglia	
				↓ Microglial phagocytosis	
	Retinal vein occlusions diabetic retinopathy retinopathy of prematurity	IR model	*None*	↑ iBRB integrity	Abcouwer et al. (2021)
				↑ Restoration of vascular barrier	
				↓ Pro-inflammatory microglia	
				↓ Retinal inflammation	
			*None*	↓ Vascular leakage	Abcouwer et al. (2013)
				↓ Retinal inflammation	
				↓ Leukocyte adhesion	
	Glaucoma	S100B	*None*	↓ Neurofilament degeneration	Grotegut et al. (2020)
				↓ Microglial activation	
				↓ Inflammatory cell infiltration	
	Experimental autoimmune uveitis	IRBP	*None*	↑ Retinal function	Zhou et al. (2020)
				↑ BRB INTEGRITY	
				↓ Microglial Activation	
				↓ Macrophage and leukocyte infiltration	
				↓ Leukocyte adhesion	
	Diabetic retinopathy	STZ	*Caspase-1/ IL-1β*	↓ Acellular capillaries	Vincent and Mohr (2007)
Minocycline			*None*	↓ Histone acetylation (AcH3K9, AcH3K18)	Wang et al. (2012)
				↓ Müller cell activation	
				↓ Retinal inflammation	
			*None*	↓ IL-1β, caspase-3 activation	Krady et al. (2005)
				↓ Retinal neuronal cell death	
				↓ Microglial cytokines	
	Age-related macular degeneration	Acute white light	*None*	↑ Photoreceptor survival	Scholz et al. (2015)
				↓ Caspase-3/7 activation	
				↓ Neurotoxicity	
				↓ Microglial activation	
				↓ Pro-inflammatory	
				↓ Retinal degeneration	
	Excitotoxicity	NMDA	*None*	↓ Microglial activation	Takeda et al. (2018)
				↓ RGC degeneration	
				↓ IL-1β, IL-6, TNF-α,	
	Glaucoma	IOP	*None*	↓ Microglial activation	Wang K. et al. (2014)
				↓ RGC survival	
				↓ CD68^+^ microglia	
	Optic glioma	Nf1^flox/flox^	*Estrogen/ ERβ*	↑ The NFL thickness	Toonen et al. (2017)
				↓ Microglial activation	

## 5. Microglia and induced pluripotent stem cell (iPSC) technology

The advent of single-cell sequencing and fate mapping techniques promises to enlarge the understanding of microglial diversity and provide a novel vision of disease-related heterogeneity and plasticity of microglia responses (Elmore et al., 2014; Huang et al., 2018a,b). However, the specific genes in microglia are instantly down-regulated when transferred from vivo to the extracorporeal culture environment (Gosselin et al., 2017). Establishing induced pluripotent stem cell-derived microglia (iMG) provides a new effective method to explore the microglial characteristic in the pathological condition, regenerative therapeutic and the intercellular co-relationship during the developing retina. iMG shows comparable signatures with purified human fetal microglia in the same culture condition which can respond to LPS and *IFN-γ* stimulation and possess professional phagocytosis (Muffat et al., 2016). The iMGs cocultured with retinal organoids furnish a foundation for future research to investigate molecular signaling mechanisms (Bartalska et al., 2022).

In self-formed ectodermal autonomous multi-zone (SEAM), a two-dimensional model of human induced pluripotent stem cells of ocular cells, iMGs enhance the expression of *VEGF-A* under the stimulus of *Tgfβ1* (Hayashi et al., 2016; Shiraki et al., 2022). Hematopoietic progenitor cells induced by pluripotent stem cells can be converted into iMGs and PSC-derived macrophages in different culture conditions; iMGs and their coculture with retinal organoids promote mutual differentiation; the differentiated iMGs migrate to the beneath photoreceptor cell layer under the coculture with retinal organoids and further differentiate into resident microglia which can promote the migration of photoreceptor precursors and may contribute to the function of pruning synapses; retinal organoids up-regulate pivotal genes which are related to development, such as *SIX3*, *SIX6*, *OTX2*, *HES1* and *DKK3* under the addition of iMGs (Gao M. L. et al., 2022). In addition, with the help of iMGs, Micklisch et al. discover that human microglia express the age-related maculopathy susceptibility 2 (*ARMS2*) transcripts which can cooperate with properdin and intensify the ability of complement activation to eliminate apoptotic and necrotic cells and its polymorphism is associated with AMD susceptibility (Micklisch et al., 2017). Similarly, iMGs, with the deficit of ADAM metallopeptidase domain 17 (*ADAM17*), present similar phenomena with *ADAM17^−/−^* Drosophila which leads to the accumulation of lipid droplet and the semblable clinical features is semblable with age-dependent degeneration of the retina (Muliyil et al., 2020).

Therefore, iMGs can be an effective reference that is vital to analyze the function of retinal microglia and potential therapeutic targets. The unique cellular properties of microglia may help retinal organoids build vascular networks.

## 6. Perspectives

Following the deep reform in single-cell RNA sequencing technology, the exploration of new targets has also been effectively explored in these years and we have generated preliminary knowledge about microglia. For example, based on previous Rna-seq sequencing data, we know the macroglial subsets specifically express the diazepam-binding inhibitor (*DBI*) which can restore microglial inflammatory response to the baseline (Menon et al., 2019). Translocator Protein *(TPSO)* in microglia is upregulated after inflammation activation. And *DBI* negatively regulates microglial activation by binding to *TSPO* and limits the extent of the inflammatory responses at the onset which facilitates the regression of inflammation (Wang M. et al., 2014). *DBI-TSPO* signaling pathway exerts anti-inflammatory and neuroprotective effects which provide clues for the research of anti-*VEGF* drugs (Gao S. et al., 2022). Although intravitreal injection of *VEGF*-inhibiting drugs is a breakthrough treatment for retinal neovascularization, the therapy targeting *VEGF* is not widely available for all patients (Ip et al., 2015). And repeated injections of anti-*VEGF* drugs are a safety concern for high-risk patients including premature infants, diabetes, and cardiovascular diseases (Usui-Ouchi and Friedlander, 2019). Based on the findings of Liu et al., there is a subset of microglia associated with neovascularization during pathological retinal angiogenesis which expresses IGF1 (Liu Z. et al., 2022). This subset may be a breakthrough to treat proliferative diabetic retinopathy and retinopathy of prematurity. *CD39*, *Tgfβ* and the renin-angiotensin system are undoubtedly potential molecular targets in terms of blocking microglia recruitment and reducing neovascularization. Hence, it is necessary to investigate the relevance of the three in retinopathy. Furthermore, we need to explore the microglial subset that induces blood vessel formation during normal retinal development, as such cells may contribute to vascularizing human retinal organoids. In addition, in the stem cell transplantation technology, the absence of microglia and chondroitin sulfate proteoglycans facilitate the migration of exogenous Müller; although the use of chondroitinase ABC and erythropoietin reduce inflammation to some extent, it still presents immune rejection at the fourth week of transplantation (Bull et al., 2008; Singhal et al., 2008). According to the RNA-seq data of Huang et al., the regenerated microglia after ablation show no significant up-regulation of inflammation-related genes (Huang et al., 2018a). Therefore, the two conjectures: stem cell transplantation technology combined with the melting regeneration properties of microglia and modified microglia for drug delivery to the retina are expected to be a promising treatment for retinal inflammation, alleviate angiogenesis and protect neurons and BRB.

Microglia have considerable potential as the only cells that can regenerate without limits in the steady-state environment of the retina. Microglia have been discovered for a century, but the mechanisms of molecular need to be further elucidated in detail.

## Author contributions

Original draft preparation was conducted by XF and SF. Writing, reviewing and editing was completed by HQ, LY, and KY. Visualization and figure creation was completed by CZ. All authors contributed to the article and approved the submitted version.

## Funding

This work was supported by National Natural Science Foundation of China (no. 31970930), Hubei Natural Science Foundation (no. 2020CFA069, no. 2018CFB434), and Neuroscience Team Development Project of Wuhan University of Science and Technology (no. 1180002).

## Conflict of interest

The authors declare that the research was conducted in the absence of any commercial or financial relationships that could be construed as a potential conflict of interest.

## Publisher’s note

All claims expressed in this article are solely those of the authors and do not necessarily represent those of their affiliated organizations, or those of the publisher, the editors and the reviewers. Any product that may be evaluated in this article, or claim that may be made by its manufacturer, is not guaranteed or endorsed by the publisher.

## Abbreviations

6-OHDA: 6-Hydroxydopamine
ACE2: Angiotensin converting enzyme 2
AMD: Age-related macular degeneration
AT1R: Angiotensin Ii type 1 receptor
AVE: Masr agonist, Ave0991
CCL5: C-C motif chemokine ligand 5
CNS: Central nervous system
CSC: Central serous chorioretinopathy
CSF-1R: Macrophage colony stimulating factor-1 receptor
CX3CR1: C-X3-C motif chemokine receptor 1
DAT: Dopamine transporters
DIZE: Diminazene aceturate Ace2 activator
DR: Diabetic retinopathy
EAU: Experimental autoimmune uveitis
ENTPD: Ectonucleoside triphosphate diphosphohydrolase
GCL: Ganglion cell layer
GSDMD: Pore-forming protein gasdermind
HIF-1Α: Hypoxia-inducible factor
IBA1: Ionized calcium binding adaptor protein 1
IFN-γ: Interferon gamma
IGF1: Insulin like growth factor 1
IL-1β: Interleukin-1β
INL: Inner nuclear layer
IOP: Intraocular pressure
IPL: Inner plexiform layer
IR: Ischemia–reperfusion injury
iBRB: Inner blood-retinal barrier
MAPK: Mitogen-activated protein kinase
MR: Mineralocorticoid receptor
NF-кB: Nuclear factor-kappa B
NMDA: N-methyl-D-aspartate
NOX: Nicotinamide adenine dinucleotide phosphate oxidase
NRF2: Nfe2-like bzip transcription factor 2
NV: Neovascularization
OIR: Oxygen-induced retinopathy
ONL: Outer nuclear layer
OPL: Outer plexiform layer
oBRB: Outer blood-retinal barrier
PD: Parkinson’s disease
PKC: Protein kinase C
PKD: Olycystic kidney disease
PP2A: Protein phosphatase 2a
RAP: Retinal angiomatous proliferation
BRB: Blood-retinal barrier
RD10: Retinitis pigmentosa
RMEC: Retinal microvascular endothelial cells
ROCK: RhoA/rhokinase
ROS: Reactive oxygen species
RPE: Retinal pigment epithelium
STAT3: Signal transducer and activator of transcription proteins 3
STZ: Streptozotocin
Tgfβ: Transforming growth factor beta
TH: Tyrosine hydroxylase
TNF-α: Tumor necrosis factor-α
TPSO: Translocator protein
VEGF: Vascular endothelial growth factor
ZO-1: Zonula ocluden-1
